# Causal relationship between beta-2 microglobulin and B-cell malignancies: genome-wide meta-analysis and a bidirectional two-sample Mendelian randomization study

**DOI:** 10.3389/fimmu.2024.1448476

**Published:** 2024-10-07

**Authors:** Jiuling Li, Yao Wu, Xin Zhang, Xueju Wang

**Affiliations:** Department of Pathology, China-Japan Union Hospital of Jilin University, Changchun, Jilin, China

**Keywords:** Beta-2 microglobulin, B-cell malignancies, causal relationship, Mendelian randomization, innate immune system

## Abstract

**Background:**

Beta-2 microglobulin (β2M) is acknowledged as a prognostic biomarker for B-cell malignancies. However, insights into the impact of β2M on B-cell malignancy risk, and vice versa, are limited.

**Methods:**

We conducted a genome-wide meta-analysis (GWMA), bidirectional two-sample Mendelian randomization (TSMR) analysis, and pathway enrichment analysis to explore the causal relationship between β2M and B-cell malignancies and the underlying biological processes.

**Results:**

The GWMA identified 55 lead SNPs across five genomic regions (three novel: WDR72, UMOD, and NLRC5) associated with β2M. In the UKB, genetically predicted β2M showed a positive association with diffuse large B-cell lymphoma (DLBCL; odds ratio [OR]: 1.742 per standard deviation increase in β2M; 95% confidence interval [CI]: 1.215–2.498; *P* = 3.00 × 10^−3^; FDR = 7.50× 10^−3^) and Hodgkin lymphoma (HL; OR: 2.270; 95% CI: 1.525–3.380; *P* = 5.15 × 10^−5^; FDR =2.58 × 10^−4^). However, no associations were found with follicular lymphoma (FL), chronic lymphoid leukemia (CLL), or multiple myeloma (MM). Reverse TSMR analysis revealed no association between genetically predicted B-cell malignancies and β2M. In FinnGen, β2M was found to be associated with an increased risk of DLBCL (OR: 2.098; 95% CI: 1.358-3.242; *P* = 8.28 × 10^−4^; FDR = 4.14 × 10^−3^), HL (OR: 1.581; 95% CI: 1.167-2.142; *P* = 3.13 × 10^−3^; FDR = 5.22 × 10^−3^), and FL (OR: 2.113; 95% CI: 1.292-3.455; *P* = 2.90 × 10^−3^; FDR = 5.22 × 10^−3^). However, no association was found with CLL or MM. Reverse TSMR analysis indicated that genetically predicted DLBCL, FL, and MM may perturb β2M levels. Pathway enrichment analysis suggested that the innate immune system represents a convergent biological process underlying β2M, DLBCL, and HL.

**Conclusions:**

Our findings suggested that elevated levels of β2M were associated with an increased risk of DLBCL and HL, which is potentially linked to dysfunction of the innate immune system.

## Graphical Abstract

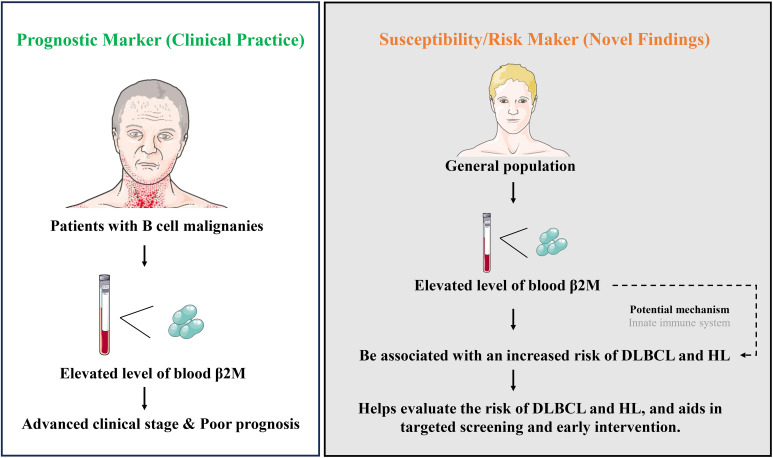

## 1 Introduction

The worldwide occurrence of B-cell malignancies is increasing annually (1, 2). Identifying risk factors improves B-cell malignancy risk assessment in the general population. Observational studies have reported that male sex, autoimmune diseases, obesity, and smoking are risk factors for B-cell malignancies (3–8). Additionally, an association between serum C-reactive protein and an increased risk of non-Hodgkin lymphoma suggested that serum biomarkers could be crucial in assessing the risk of B-cell malignancies (9).

Beta-2 microglobulin (β2M) is a component of the major histocompatibility complex (MHC) class I molecule, which is present on the surface of almost all nucleated cells. Blood levels of β2M may have varied clinical implications as a biomarker. β2M is a prognostic marker in patients with B-cell malignancies. Observational studies have shown that β2M is independently associated with poor survival in patients with various B-cell malignancies, including diffuse large B-cell lymphoma (DLBCL; hazard ratio [HR]: 2.9-6.5) (10, 11), Hodgkin’s lymphoma (HL; 5-7 year overall survival rates were 52%-73%) (12, 13), follicular lymphoma (FL; HR: 2.9) (14), chronic lymphocytic leukemia (CLL; HR: 1.2-2.3) (15–17), and multiple myeloma (MM; HR: 1.8) (18). Despite evidence of elevated β2M levels in B-cell malignancies, no studies have yet published findings on the association between β2M and the risk of B-cell malignancies in the general population.

Due to confounding factors and reverse causation in observational studies, determining whether β2M influences the risk of developing B-cell malignancies or vice versa is challenging. Mendelian randomization (MR) analysis identifies causal relationships between risk factors and outcomes by using genetic variants, thereby avoiding confounding factors and reverse causation (19–21). In 2020, Kleinstern et al. used MR analysis and found no causal relationship between lipid traits and non-Hodgkin lymphoma (22). Recently, through MR analysis, Wang et al. reported that inflammatory factors, including interleukin-7 and interleukin-10, were associated with an increased risk of MM (23). In summary, MR analysis serves as a vital tool for assessing the causal effects of β2M on the risk of B-cell malignancy and vice versa.

This study conducted a genome-wide meta-analysis (GWMA) of β2M with 40,927 Europeans, identifying additional novel loci for β2M. Using the expanded list of genetic risk alleles as an instrument for identifying β2M, we conducted further bidirectional two-sample MR (TSMR) analyses between β2M and B-cell malignancies (DLBCL, FL, HL, CLL, and MM). Additionally, we performed pathway enrichment analysis for genes overlapping between β2M and B-cell malignancies. We identified three novel loci associated with β2M and observed that elevated β2M was associated with an increased risk of DLBCL and HL, potentially due to dysfunction of the innate immune system.

## 2 Materials and methods

### 2.1 Study design

**Figure 1:**
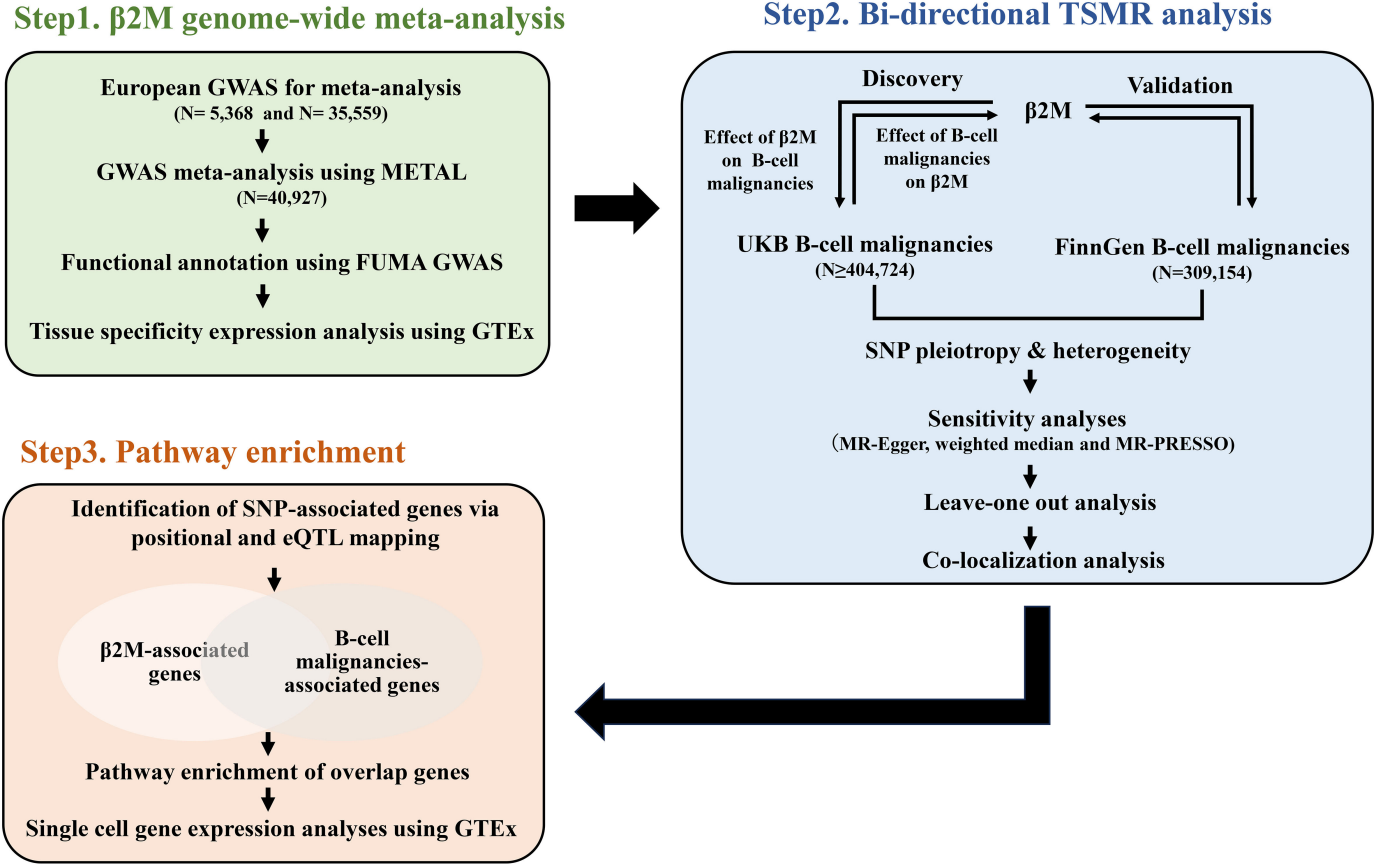
A schematic diagram of the study design. Briefly, a GWMA was conducted to detect loci associated with β2M (Step1). SNPs significantly and independently associated with the exposure (β2M/B-cell malignancies) were subjected to TSMR to identify the causal relationship between β2M and B-cell malignancies (Step2). Finally, pathway enrichment analysis was utilized to explore the underlying biological processes linking β2M and B-cell malignancies (Step3). β2M, beta-2 microglobulin; CLL, chronic lymphoid leukemia; DLBCL, diffuse large B-cell lymphoma; eQTL, expression quantitative trait locus; FL, follicular lymphoma; HL, Hodgkin lymphoma; GWAS, genome-wide association study; GWMA, genome-wide meta-analysis; MM, multiple myeloma; TSMR, two-sample Mendelian randomization; UKB, UK Biobank.

As shown in **Figure 1**, we first conducted a genome-wide meta-analysis (GWMA) of β2M by combining a GWAS from the GWAS Catalog and deCODE. This combination resulted in a total sample size of 40,912. Functional annotation of the GWMA summary was performed using the Functional Mapping and Annotation of Genome-Wide Association Studies (FUMA GWAS) web-based tool. Tissue specificity expression analysis was performed using the “Gene Page” of the Genotype-Tissue Expression (GTEx) portal. To investigate the causal relationship between β2M and B-cell malignancies (DLBCL, FL, HL, CLL and MM), we performed a bidirectional TSMR analysis. Briefly, we conducted forward and reverse TSMR analyses to explore the causal relationship between β2M levels and B-cell malignancies using GWMA summary statistics for β2M levels and UK Biobank (UKB) GWAS data for B-cell malignancies. Additionally, we used FinnGen GWAS data on B-cell malignancies for bidirectional TSMR to validate the causal association. Furthermore, we conducted pathway enrichment analysis using overlapping genes between β2M and B-cell malignancies to investigate the convergence of biological processes underlying β2M and B-cell malignancies.

### 2.2 Data sources

Similar genetic variant-phenotype associations in separate samples are the basic assumption of TSMR. This assumption may be violated if samples are from different populations (e.g., populations of different ages, sexes or ethnicities). To hold the assumption, we selected β2M and B-cell malignancy GWASs in which participants were mainly of European ancestry for both sexes. The detailed GWAS summary statistics used for the TSMR analyses are described in **Supplementary Table S1**. Briefly, the GWAS summary statistics of β2M were obtained from two published studies on GWAS of plasma or serum protein levels involving 35,559 and 5,353 individuals of European descent, respectively (24, 25). The GWAS summary statistics for B-cell malignancies were obtained from the UKB and FinnGen consortia. The UKB cohort included over 500,000 adult individuals recruited from the UK population between 2006 and 2010. Lee et al. analyzed ~1,400 binary phenotypes in 400,000 white British participants, including DLBCL (573 patients and 404,466 controls), FL (371 patients and 404,466 controls), HL (258 patients and 404,466 controls), CLL (506 patients and 404,466 controls) and MM (552 patients and 361,060 controls) patients. All analysis summary statistics are available from https://www.leelabsg.org/resources. FinnGen collects and analyses genome and phenotypic data from hundreds of thousands (aiming at 500,000 individuals in the end of 2023) of Finnish biobank participants. For FinnGen release 7, a total of 309,154 participants were evaluated for 3,095 phenotypes, including DLBCL (352 patients and 308,802 controls), FL (816 patients and 308,338 controls), HL (586 patients and 308,568 controls), CLL (437 patients and 308,717 controls), and MM (914 patients and 308,240 controls). All analysis summary statistics are available from https://www.finngen.fi/en/access results.

The β2M assessment method and diagnostic criteria for B-cell malignancy are available in **Supplementary Table S1**.

### 2.3 β2M genome-wide meta-analysis

We searched for publicly available β2M GWASs before November 4, 2023, with “beta-2-microglobulin”, “β2M”, and “B2M” as keywords in the GWAS Catalog. We found that 5 GWASs on β2M have been published, but summary statistics are available for only 1 GWAS (N=5,353), which focused on European populations (**Supplementary Table S2**) (24). DeCODE genetics has also published a GWAS on β2M involving 35,559 individuals (**Supplementary Table S2**) (25). We performed a genome-wide meta-analysis (GWMA) combining these 2 independent GWASs focused on β2M, encompassing a total of 40,912 subjects. GWMA was performed using a fixed-effect inverse variance–weighted model with METAL. (26) The autosomal SNPs that showed the same direction of effect and had a P value for heterogeneity greater than 0.05 across the 2 GWASs were utilized in subsequent research.

### 2.4 Functional annotation of genome-wide meta-analysis

We employed the web-based tool FUMA GWAS to identify genomic risk loci and to acquire functional information about the relevant SNPs within these loci (27). First, lead SNPs were defined using a genome-wide significant P value (5×10^−8^) and linkage disequilibrium (LD) r^2^ <0.05. All SNPs with a significant P value (0.05) in LD (r^2^ ≥0.05) with one of the lead SNPs were candidate SNPs. Furthermore, genomic risk loci were identified by merging LD blocks that were less than 250 kb apart.

Gene mapping was based on positional mapping and expression quantitative trait locus (eQTL) mapping. First, positional mapping was performed by applying a maximum distance of 10 kb between SNPs and genes. Second, eQTL mapping was conducted using data generated in GTEx v8. The Benjamini-Hochberg false discovery rate (FDR) method, with a threshold of 0.05, was employed to define significant eQTL associations. Tissue specificity expression analysis was performed using the “Gene Page” of the Genotype-Tissue Expression (GTEx) portal.

### 2.5 Bidirectional TSMR analysis

MR analysis was based on three core assumptions: (1) relevance: single-nucleotide polymorphisms (SNPs) are associated with exposure; (2) independence: SNPs are not associated with confounders; and (3) exclusion restriction: SNPs affect the outcome only through the exposure. The SNPs satisfying these assumptions are known as valid instrumental variables (IVs).

In this study, SNPs significantly and independently associated with exposure were selected as IVs. Briefly, we used significant, independent lead SNPs in GWMA as IVs to proxy β2M levels. We chose SNPs under a lenient threshold of *P* < 1× 10^−5^ to predict B-cell malignancies because few SNPs with *P* < 5 × 10^−8^ were available. To ensure that these SNPs were independent of each other, we pruned them based on LD (*r* ^2^ < 0.05; within a 10 Mb distance).

We performed the primary analysis via an inverse variance-weighted (IVW) approach, which assumes that all SNPs are valid IVs (28). MR-Egger regression and Cochran’s Q test were used to estimate SNP pleiotropy and heterogeneity effects. The estimates were considered robust when *P* > 0.05 for both the regression intercept and Q test. However, the pleiotropy effects of IVs are common and difficult to avoid. To minimize the bias of the pleiotropy effects, we conducted sensitivity analyses using MR-Egger (29), weighted median (30) and the Mendelian randomization pleiotropy residual sum and outlier (MR-PRESSO) (31) approach, as detailed in our previous study (32). In addition, leave-one-out analysis was used to identify potential influential SNPs that may drive causal associations. The TSMR analysis was conducted using the “TwoSampleMR” package of R (version 0.5.6). A false discovery rate (FDR) of 0.05 was used to define significant associations.

### 2.6 Colocalization analysis

To detect whether the identified causal associations are driven by causal variants that are in linkage disequilibrium (LD) within a genomic region, we further performed colocalization analyses using the ‘coloc’ package (https://github.com/chr1swallace/coloc) to detect shared causal variants for the significant associations in the TSMR analyses. We focused on the genomic region within 5 kb on both sides of the IVs used in the TSMR analyses. We utilized the coloc.abf algorithm and identified SNPs within the region with a posterior probability greater than 0.95 as candidate causal variants. Those candidates showing LD *r^2^* > 0.8 with IVs and an association P value < 0.05 were determined to be causal variants.

### 2.7 Pathway enrichment

To investigate the convergence of biological processes underlying β2M and B-cell malignancies, we performed pathway enrichment analysis on overlapping genes between β2M and five B-cell malignancies. B-cell malignancy GWAS SNP-associated genes were identified based on positional and eQTL mapping, as mentioned above. Pathway enrichment analysis of the overlapping genes was conducted using the ‘gene2func’ module in the FUMA GWAS. The pathways used in this study were derived from GO biological processes, KEGG, and canonical pathways of the MsigDB C2 collection. Single-cell gene expression analyses were performed using the “Multi Gene Single-Cell Query Page” of the GTEx Portal. The TIMER2.0 online database was used to investigate the relationships between the NLRC5 expression level and B2M and innate immune system genes in DLBCL (33).

## 3 Results

### 3.1 Identification and functional annotation of genetic loci associated with β2M in GWMA

**Figure 2:**
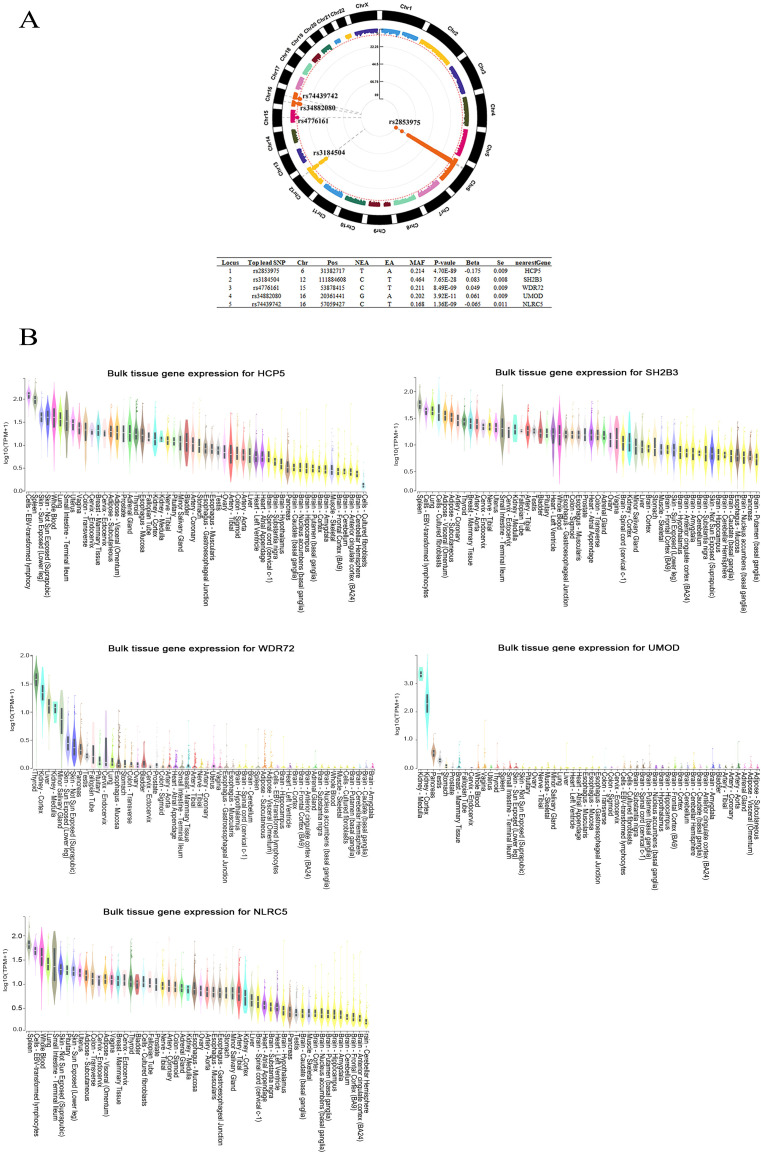
The genome-wide meta-analysis of β2M. **(A)** Five loci associated with β2M are shown in the Manhattan plot. **(B)** Bulk tissue gene expression analysis of the genes associated with the 5 top SNPs. HCP5, SH2B3, and NLRC5 were found to be highly expressed in EBV-transformed lymphocytes, as well as in lymphocyte-rich whole blood and spleen tissues. High expression levels of WDR72 and UMOD were observed in kidney tissues. Beta, regression coefficient; Chr, chromosome; EA, effect allele; Locus, Index of genomic risk loci; MAF, minor allele frequency; NEA, noneffect allele; nearestGene, the nearest gene of the SNP based on ANNOVAR annotations; Pos, position of top lead SNP based on the human genome build hg19; P value, the p value of the association; SE, standard error of Beta; Top lead SNP, lead SNP that has the most significant P value in the locus.

The GWMAs of 2 independent GWASs on β2M revealed 5 genetic loci associated with β2M (**Figure 2A**). Of the 5 identified loci, 2, rs2853975 in HCP5 (HLA complex P5) and rs3184504 in SH2B3 (SH2B adaptor protein 3), were previously reported by Tin A et al. (34) The 3 newly identified loci were rs4776161 in WDR72 (WD repeat domain 72), rs34882080 in UMOD (Uromodulin), and rs74439742 in NLRC5 (NLR family CARD domain containing 5). HCP5, SH2B3, and NLRC5 were highly expressed in EBV-transformed lymphocytes, as well as in whole blood and spleen tissues enriched with lymphocytes. In contrast, WDR72 and UMOD exhibited high expression levels in kidney tissues (**Figure 2B**). There were 55 lead SNPs, with 25 associated with higher β2M levels and 30 associated with lower β2M levels (**Supplementary Table S3**), along with 21,025 candidate SNPs located within these 5 loci (**Supplementary Table S4**). The majority of these SNPs are located in intergenic and intronic regions (**Supplementary Figure S1**). A total of 526 genes mapped to SNPs associated with β2M were identified through positional mapping and eQTL mapping, and these genes were subsequently used for pathway enrichment analysis (**Supplementary Table S5**).

### 3.2 Discovery of associations between β2M and B-cell malignancies in the UKB

Initially, we conducted forward Mendelian randomization (MR) analyses using 55 lead SNPs associated with β2M levels and UKB GWAS data for B-cell malignancies to explore the causal effects of genetically predicted β2M levels on B-cell malignancies.

**Figure 3:**
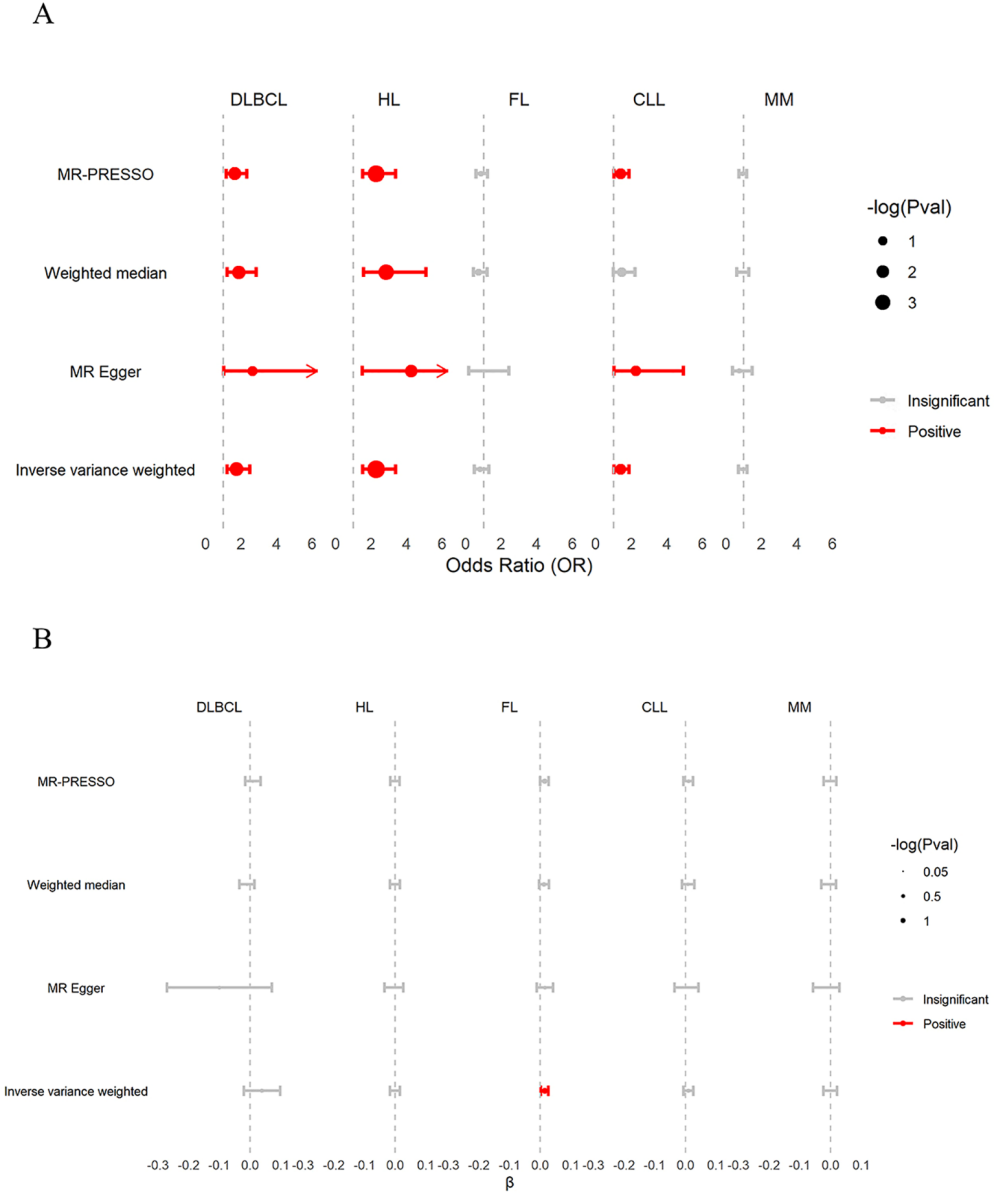
Discovery of associations between β2M and B-cell malignancies in the UKB. **(A)** The causal effects of genetically predicted β2M on B-cell malignancies. β2M was found to be positively associated with DLBCL, HL, and CLL, but not with FL and MM. **(B)** The causal effects of genetically predicted B-cell malignancies on β2M. DLBCL, HL, CLL, and MM were not associated with β2M. However, inverse variance weighting (IVW) analysis showed that FL was positively associated with β2M levels. β, effect size of B-cell malignancies on β2M; CLL, chronic lymphoid leukemia; DLBCL, diffuse large B-cell lymphoma; FL, follicular lymphoma; HL, Hodgkin lymphoma; MM, multiple myeloma.

As shown in **Figure 3A** and **Supplementary Table S6**. The IVW estimates showed that β2M was positively associated with DLBCL (odds ratio [OR]: 1.742 per standard deviation increase in β2M; 95% confidence interval [CI]: 1.215–2.498; *P* = 3.00 × 10^−3^; FDR = 7.50× 10^−3^), HL (OR: 2.270; 95% CI: 1.525-3.380; *P* = 5.15 × 10^−5^; FDR =2.58 × 10^−4^), and CLL (OR: 1.387; 95% CI: 1.032-1.864; *P* = 3.00×10^−2^; FDR = 5.00×10^−2^) but was not associated with FL (OR: 0.785; 95% CI: 0.476-1.294; *P* = 0.255; FDR =0.342) or MM (OR: 0.913; 95% CI: 0.706-1.180; *P* = 0.131; FDR =0.486). Cochran’s Q test revealed heterogeneity in the associations of β2M with DLBCL (*P* = 1.37 × 10^−5^) and FL (*P* = 1.06 × 10^−8^). MR-PRESSO identified one outlier SNP in the association between β2M and DLBCL, indicating that the corrected causal relationship was still significant. Furthermore, three outlier SNPs were identified in the association between β2M and FL, with the corrected causal relationship remaining insignificant. The results from weighted median estimates or MR-Egger were consistent with the IVW estimation results. The leave-one-out analysis (**Supplementary Figure S2D**) demonstrated that the association between β2M and CLL was driven by potentially influential SNPs. After removing potentially influential SNPs, the association between β2M and CLL (OR: 1.139; 95% CI: 0.837-1.549; P = 0.409) was found to be nonsignificant. Colocalization analyses revealed that the associations between β2M and DLBCL, between β2M and HL, and between β2M and CLL may be attributed to causal SNPs (**Supplementary Table S7**). After removing causal SNPs, significant associations remained between β2M and DLBCL (OR: 1.477; 95% CI: 1.000-2.183; P = 5.00×10^−2^) and between β2M and HL (OR: 2.167; 95% CI: 1.430-3.284; P = 2.67×10^−4^), but the association with CLL was not significant (OR: 1.262; 95% CI: 0.938-1.699; P = 0.124). These findings suggested that elevated β2M was associated with an increased risk of DLBCL and HL but not FL, CLL, or MM.

As shown in **Figure 3B** and **Supplementary Table S6**, the IVW estimates of the reverse TSMR analysis on the effects of B-cell malignancies on β2M revealed that FL showed a positive association with β2M (β: 0.014; 95% CI: 0.002–0.026; *P* = 0.029; FDR =0.145), although the association did not remain significant after FDR correction. Neither DLBCL (β: 0.039; 95% CI: -0.020–0.098; *P* = 0.188; FDR =0.400), HL (β: -0.001; 95% CI: -0.017–0.015; *P* = 0.856; FDR=0.898), CLL (β: 0.010; 95% CI: -0.006–0.026; *P* = 0.240; FDR = 0.400), nor MM (β: -0.001; 95% CI: -0.023–0.021; *P* = 0.898; FDR = 0.898) were associated with β2M. Cochran’s Q test revealed heterogeneity in the associations of β2M with DLBCL (*P* = 8.69 × 10^−24^) and CLL (*P* = 1.20 × 10^−2^). MR-PRESSO identified four outlier SNPs in the association between DLBCL and β2M, with the corrected causal relationship remaining insignificant. Furthermore, no outlier SNPs were detected in the association between CLL and β2M. The results from weighted median estimates or MR-Egger were consistent with the IVW estimation results. The leave-one-out analysis (**Supplementary Figure S3C**) demonstrated that the causality between FL and β2M was driven by potentially influential SNPs. After removing potentially influential SNPs, the association between FL and β2M (β: 0.001; 95% CI: -0.011–0.014; *P* = 0.821) was not significant. These findings suggested that B-cell malignancies do not affect β2M levels.

The associations of IVs with β2M and B-cell malignancies are available in **Supplementary Table S8**.

### 3.3 Validation of the associations between β2M and B-cell malignancies in FinnGen

We performed TSMR analyses based on FinnGen to validate the causal association between β2M and B-cell malignancies.

**Figure 4:**
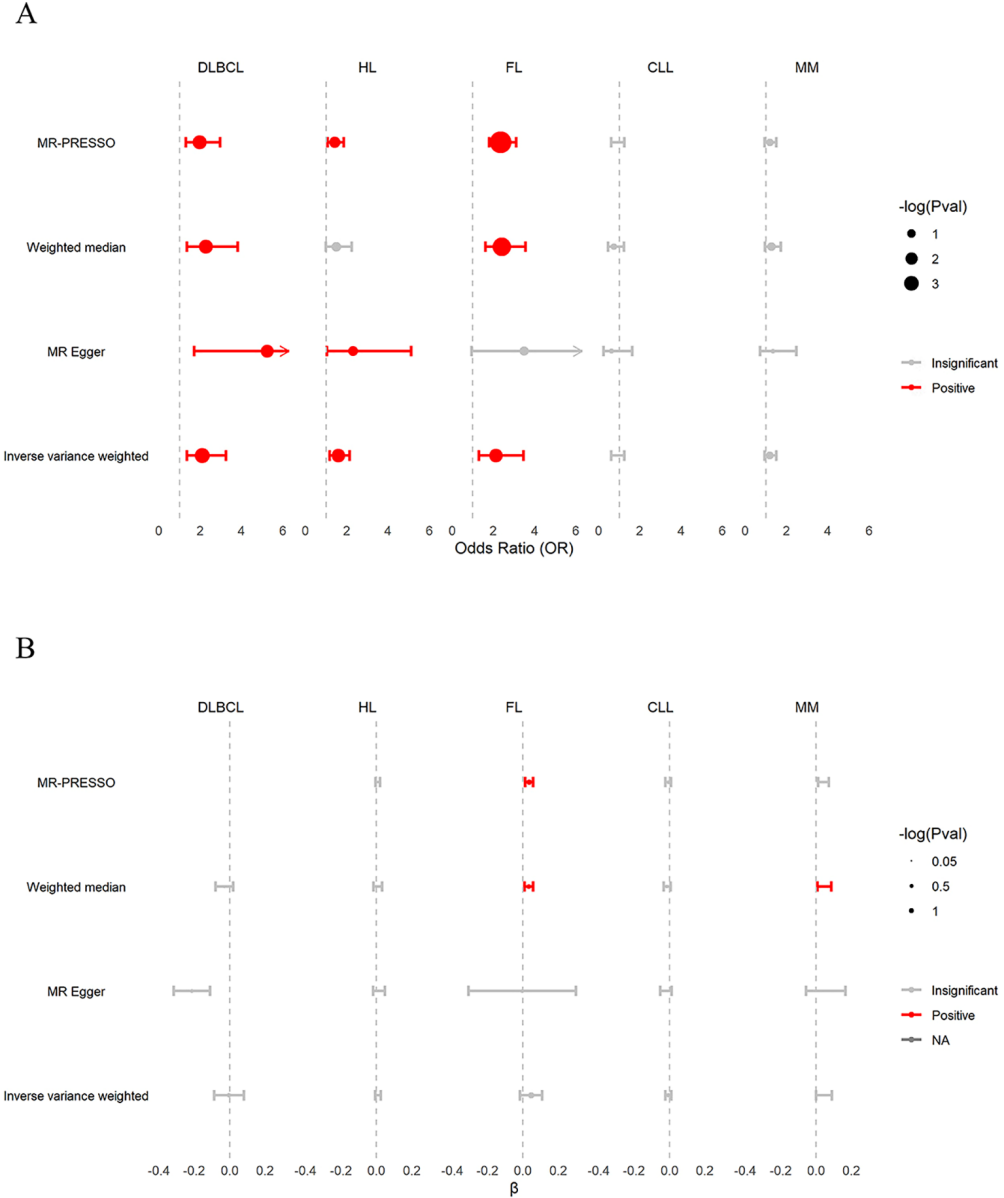
Validation of associations between β2M and B-cell malignancies in FinnGen. **(A)** The causal effects of β2M on B-cell malignancies. β2M was positively associated with DLBCL, HL, and FL, but not with CLL and MM. **(B)** The causal effects of B-cell malignancies on β2M. DLBCL, HL, FL, CLL, and MM were not associated with β2M. β, effect size of B-cell malignancies on β2M; CLL, chronic lymphoid leukemia; DLBCL, diffuse large B-cell lymphoma; FL, follicular lymphoma; HL, Hodgkin lymphoma; MM, multiple myeloma.

As shown in **Figure 4A** and **Supplementary Table S9**. The IVW estimates replicated the TSMR results on the causal effects of genetically predicted β2M on B-cell malignancies, confirming that β2M was associated with increased risk of DLBCL (OR: 2.098; 95% CI: 1.358-3.242; *P* = 8.28 × 10^−4^; FDR = 4.14 × 10^−3^) and HL (OR: 1.581; 95% CI: 1.167-2.142; *P* = 3.13 × 10^−3^; FDR = 5.22 × 10^−3^) but was not associated with CLL (OR: 0.870; 95% CI: 0.608-1.246; *P* = 0.448; FDR = 0.448) or MM (OR: 1.189; 95% CI: 0.943-1.498; *P* = 0.144; FDR = 0.180). Contrary to the UKB results, the FinnGen findings suggested that β2M was also positively associated with FL (OR: 2.113; 95% CI: 1.292-3.455; *P* = 2.90 × 10^−3^; FDR = 5.22 × 10^−3^). Cochran’s Q test revealed heterogeneity in the associations of β2M with DLBCL (*P* = 2.00× 10^−3^), HL (*P* = 4.40 × 10^−2^), FL (*P* = 9.92 × 10^−28^) and CLL (*P* = 2.70× 10^−2^). MR-PRESSO identified one, one, and six outlier SNPs in the association of β2M with DLBCL, HL, and FL, respectively, and the corrected causal relationships were still significant. Furthermore, no outliers were detected in the association between β2M and CLL. The results from weighted median estimates or MR-Egger were consistent with the IVW estimation results. Plots of the leave-one-out analysis (**Supplementary Figure S4**) demonstrated that there were no potentially influential SNPs driving the causal association. Colocalization analyses revealed that the associations between β2M and DLBCL and between β2M and FL may be attributed to causal SNPs (**Supplementary Table S10**). After removing causal SNPs, significant associations remained between β2M and DLBCL (OR: 1.946; 95% CI: 1.242-3.049; *P* = 3.68×10^−3^) and between β2M and FL (OR: 1.805; 95% CI: 1.096-2.972; *P* = 2.03×10^−2^). These findings suggested that elevated β2M was associated with an increased risk of DLBCL, HL, and FL but not CLL or MM.

As shown in **Figure 4B** and **Supplementary Table S9**, the reverse TSMR analysis of the effects of B-cell malignancies on β2M showed a consistent association, as noted in the UKB findings. Briefly, the IVW estimates showed that DLBCL (β:-0.003; 95% CI: -0.087–0.081; *P* = 0.936; FDR = 0.936), HL (β: 0.009; 95% CI: -0.007–0.025; *P* = 0.314; FDR = 0.523), FL (β: 0.046; 95% CI: -0.017–0.109; *P* = 0.148; FDR = 0.370), CLL (β: -0.006; 95% CI: -0.022–0.010; *P* = 0.427; FDR = 0.534) and MM (β: 0.045; 95% CI: 0.000–0.090; *P* = 5.00 × 10^−2^; FDR = 0.250) were not associated with β2M. Cochran’s Q test revealed heterogeneity in the associations of DLBCL (*P* = 1.16× 10^−4^), FL (*P* = 1.50 × 10^−70^), and MM (*P* = 1.00 × 10^−3^) with β2M. The effects of DLBCL on β2M could not be inferred using MR-PRESSO due to an insufficient number of SNPs. In the association between FL and β2M, MR-PRESSO identified seven outlier SNPs, and the corrected causal relationship was significant. In the association between MM and β2M, MR-PRESSO identified two outlier SNPs, and the corrected causal relationship remained insignificant. The results from weighted median estimates or MR-Egger were consistent with the IVW estimation results. Leave-one-out analysis (**Supplementary Figure S5A, C, E**) demonstrated that the associations of DLBCL, FL, and MM with β2M were driven by potentially influential SNPs. After removing potentially influential SNPs, DLBCL was negatively associated with β2M (β: -0.049; 95% CI: -0.085–0.014; *P* =6.12 × 10^−3^). In contrast, FL (β: 0.060; 95% CI: 0.003–0.118; *P* = 0.038) and MM (β: 0.077; 95% CI: 0.049–0.105; *P* = 5.30 × 10^−8^) showed positive associations with β2M. These findings suggest that DLBCL, FL, and MM may affect β2M levels.

The associations of IVs with β2M and B-cell malignancies are available in **Supplementary Table S11**.

### 3.4 Potentially biological mechanisms underlying the role of β2M in DLBCL and HL

**Figure 5:**
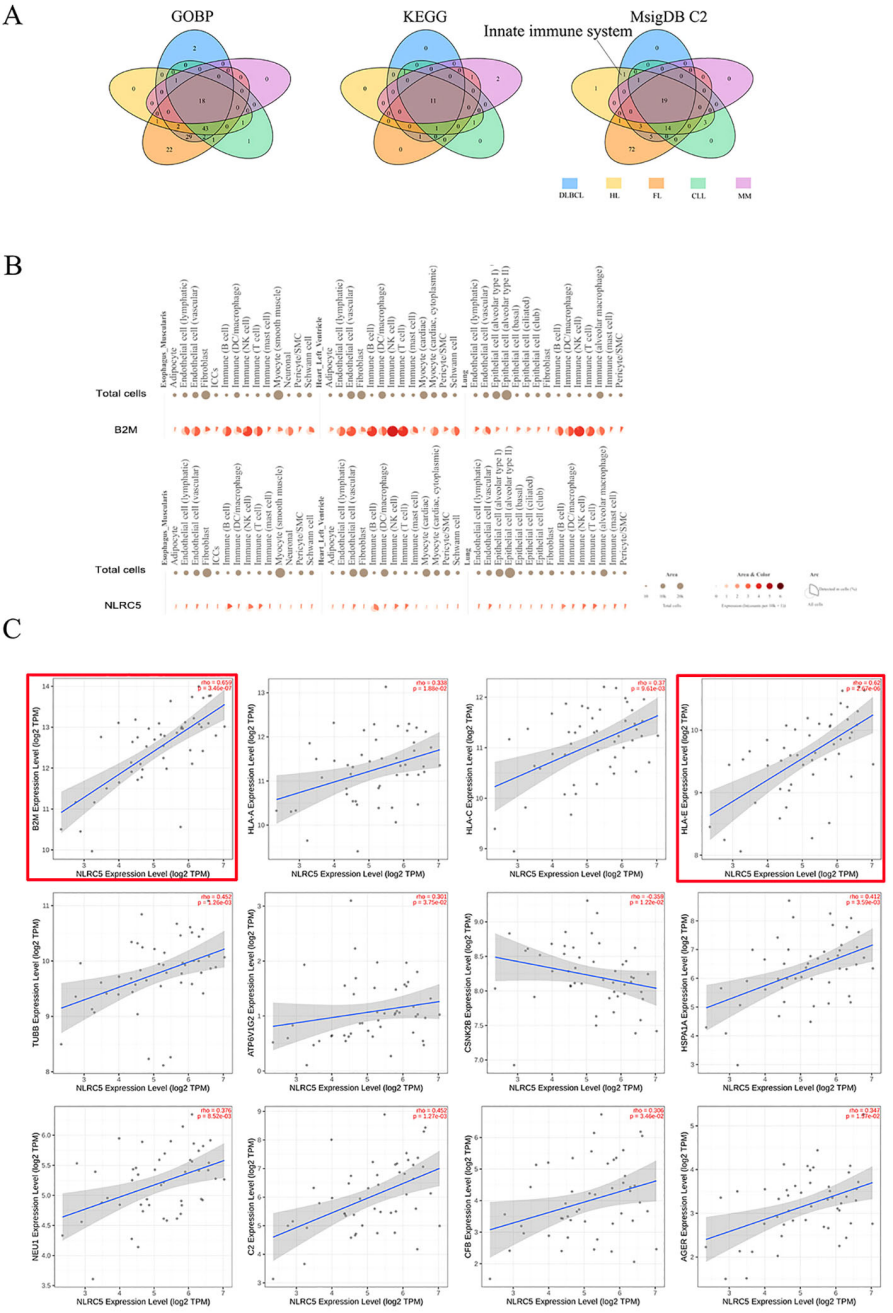
Potentially biological mechanisms underlying the role of β2M in DLBCL and HL. **(A)** The innate immune system is the pathway uniquely shared by β2M, DLBCL, and HL, as illustrated in the Venn diagram. **(B)** Single-cell gene expression analyses of B2M and NLRC5 in typical representative tissues. B2M was highly upregulated in innate immune cells, particularly in natural killer cells. A similar upregulation pattern was observed for the B2M transcription factor NLRC5. **(C)** Correlation analysis of NLRC5 with B2M and innate immune system genes. NLRC5 exhibited a strong correlation with both B2M and HLA-E (r > 0.6), as indicated by the red boxes.

We identified 525, 316, 193, 477, 303, and 166 genes associated with β2M, DLBCL, HL, FL, CLL, and MM, respectively, as listed in **Supplementary Table S12**. Pathway enrichment analysis was conducted on genes overlapping between β2M and DLBCL (228 genes), HL (120), FL (337), CLL (130), and MM (38). As shown in **Figure 5A** and detailed in **Supplementary Table S13**, the GOBP and KEGG analyses did not identify specific pathways for DLBCL and HL. However, analysis of the canonical pathways from the MsigDB C2 collection revealed that the innate immune system was more specific for DLBCL and HL than for FL, CLL, and MM, involving 17 genes, including MHC class I molecules (HLA-A, B,C, and E), complement system (C2, CFB, C4A, and C4B), heat shock protein family A (HSPA1A and HSPA1B), proteasome 20S subunit beta (PSMB8 and PSMB9), tubulin beta class I (TUBB), ATPase H+ transporting V1 subunit G2 (ATP6V1G2), casein kinase 2 beta (CSNK2B), neuraminidase 1(NEU1), and advanced glycosylation end-product specific receptor (AGER). This finding suggested that the innate immune system is a convergent biological process underlying β2M, DLBCL, and HL. **Figure 5B** shows that B2M was highly upregulated in innate immune cells within typical representative tissues, particularly in natural killer cells. A similar upregulation pattern was observed for the B2M transcription factor NLRC5. This pattern was also evident in other tissue types not displayed, such as esophageal mucosa, skeletal muscle, and prostate. Furthermore, in DLBCL patient tumor tissues, NLRC5 showed significant associations not only with B2M but also with 11 genes involved in the innate immune system (**Figure 5C**). Among these genes, NLRC5 had a strong correlation with B2M and HLA-E (rho>0.6).

## 4 Discussion

We conducted a GWMA on β2M and identified three novel loci associated with β2M levels: WDR72, UMOD, and NLRC5. WDR72 and UMOD are highly expressed in kidney tissues, while NLRC5 is highly expressed in EBV-transformed lymphocytes, whole blood and spleen tissues. For the first time, we conducted bidirectional TSMR analyses to assess the causal relationship between β2M and B-cell malignancies. Both the UKB and FinnGen forward TSMR analyses provided clear evidence of a risk-increasing effect of β2M on DLBCL and HL, without supporting evidence for its causal effect on CLL and MM. Additionally, reverse TSMR analyses from both UKB and FinnGen showed that HL and CLL did not affect β2M levels. Finally, we identified the innate immune system as a convergent biological process underlying β2M, DLBCL, and HL.

We identified three novel loci (WDR72, UMOD, and NLRC5) associated with β2M levels. WDR72 and UMOD are highly expressed in the kidney, while NLRC5 is predominantly expressed in lymphocyte-enriched tissues. β2M freely passes through the glomerular filtration membrane, with 99.9% of the filtered β2M being reabsorbed by proximal convoluted tubule cells and subsequently degraded into amino acids, preventing its re-entry into the bloodstream (35). The rs4776161-T allele in WDR72 and rs34882080-A allele in UMOD were both associated with increased β2M levels. However, the rs17730281-G allele, which is in strong LD with rs4776161-T (r2>0.8), was negatively associated with log-transformed eGFR creatinine levels, and rs34882080-A was associated with a reduced glomerular filtration rate (36, 37). These observations indicate that impaired kidney function, particularly glomerular filtration dysfunction, may contribute to elevated β2M levels. Additionally, NLRC5 is highly expressed in lymphocyte-enriched tissues and serves as a key transcriptional regulator of B2M and MHC class I genes (HLA-A, B, C, and E) (38, 39). Previous study has reported that the rs74439742 in NLRC5 is associated with a reduced HLA-E/LILRB1 protein level ratio (40). Our study has further identified a novel association between rs74439742-T and reduced β2M levels. These findings suggest that rs74439742 may affect NLRC5 function, leading to changes in the expression of HLA-E and B2M. These findings suggest that β2M levels are influenced by two key factors: glomerular filtration function and protein synthesis. While our study has identified novel genetic loci significantly associated with β2M, these findings necessitate validation through larger-sample GWAS to ensure their robustness and generalizability.

β2M is a well-recognized prognostic biomarker in various B-cell malignancies, including DLBCL, HL, FL, MM, and CLL, and it is even recommended by the International Staging System for stratifying MM patients (10–18). Furthermore, our study found that β2M may be a susceptibility or risk factor for DLBCL and HL. This suggests that β2M serves dual roles: it is a prognostic marker in already diagnosed patients and also potential role as a susceptibility or risk factor in the general population. In the context of MM, β2M may not be involved in the early onset of the disease but rather in its progression once the disease has been established. This distinction is crucial and may also be relevant for patients with CLL. Conversely, in DLBCL and HL, β2M appears to play a role not only in the early onset of these diseases but also in regulating their progression. Additionally, our reverse TSMR analysis, based on UKB and FinnGen, showed no evidence of a causal effect of HL or CLL on β2M levels, and the impact of DLBCL, FL, and MM on β2M levels remains controversial. We cannot rule out the potential influence of these malignancies on β2M levels, and further research is necessary to clarify these potential associations.

β2M, found on tumor cell membranes, is known to bind noncovalently to human leukocyte antigen-I and participate in immune regulation by forming MHC-I complexes (41). Patients with DLBCL often exhibit inactivating mutations and focal deletions in β2M, leading to the inhibition of MHC class I molecules (HLA-A,B, and C) expression on the cell surface. The reduction in MHC class I complex expression facilitates immune escape by tumor cells, thereby contributing to tumorigenesis (42). Of interest, the HLA-E/β2M heterodimers can suppress the immune effector functions of NK cells and T cells by binding to the CD94/NKG2A receptor on these cells, potentially leading to their functional exhaustion. This suppression significantly weakens the cytotoxic capabilities of NK cells and T cells, thereby facilitating immune evasion by tumor cells (43). NLRC5, as a transcription factor, regulates the expression of MHC class I genes and B2M. Our study found that the missense mutation rs74439742-T in the NLRC5 is associated with lower β2M levels, while rs74439742-C is associated with higher β2M levels. Additionally, UKB and FinnGen GWAS showed that the rs74439742-T variant exhibits a trend of negative correlation with DLBCL (P_FinGen_=0.302 and P_UKB_=0.262). These findings suggest that NLRC5 carrying the rs74439742-C allele may play an oncogenic role in the development of DLBCL, potentially through the elevation of β2M levels. However, the detailed mechanisms underlying this association require further investigation.

The immunoregulatory potential of circulating β2M remains relatively underexplored. Our findings reveal that the biological processes associated with β2M levels, DLBCL, and HL are closely linked to the innate immune system, highlighting the significance of the tumor immune microenvironment (TIME). It has been reported that daily intravenous injections of recombinant human β2M in mice for 3 days increased the percentage of proinflammatory monocytes in the plasma, which produce inflammatory factors such as C-X-C motif chemokine ligand 1 (CXCL1), tumor necrosis factor (TNF-α), and matrix metallopeptidase 9 (MMP9) (44). Further research is needed to determine whether these proinflammatory monocytes and their associated cytokines contribute to the pathogenesis of DLBCL and HL. Histopathologically, DLBCL and HL are characterized by a highly diverse and plastic TIME, typically consisting of various immune cell types. In contrast, MM and CLL are predominantly marked by the monoclonal proliferation of tumor cells with a deficiency in immune cell infiltration. This distinction may explain the observed association between elevated β2M levels and an increased risk of DLBCL and HL, an association not observed in CLL and MM. Future research should explore the relationship between β2M levels and clinicopathological features across these malignancies, with particular attention to immune cells within TIME, such as T cells and M2 macrophages. This could provide valuable insights into the biological mechanisms that define β2M’s role as either a prognostic marker in diagnosed patients or a risk factor in the general population.

Some limitations of the current work should be acknowledged. First, although TSMR provides valuable insights into causal relationships, it is subject to inherent limitations. Specifically, the assumptions of independence and exclusion restriction not always be met, which may impact the validity of the findings. Therefore, further validation through prospective studies or randomized controlled trials (RCTs) is recommended. Moreover, the GWASs utilized in this study primarily involved populations of European descent. In the absence of available β2M GWAS data for Asian populations, prospective studies in these groups are essential to validate our findings and determine their generalizability across different ethnic groups.

## 5 Conclusion

This study identified novel loci associated with β2M, confirming its role as a susceptibility or risk marker for DLBCL and HL, as well as its underlying biological mechanisms. These findings can help in assessing the risk of DLBCL and HL, allowing for more targeted screening and early intervention.

## Data Availability

The original contributions presented in the study are included in the article/**Supplementary Material**. Further inquiries can be directed to the corresponding author.
