# Supplementary figures and images for: Causal relationship between beta-2 microglobulin and B-cell malignancies: genome-wide meta-analysis and a bidirectional two-sample Mendelian randomization study
Source: Front Immunol. 2024 Oct 7;15:1448476. doi: 10.3389/fimmu.2024.1448476 (PMC11491367; doi:10.3389/fimmu.2024.1448476)

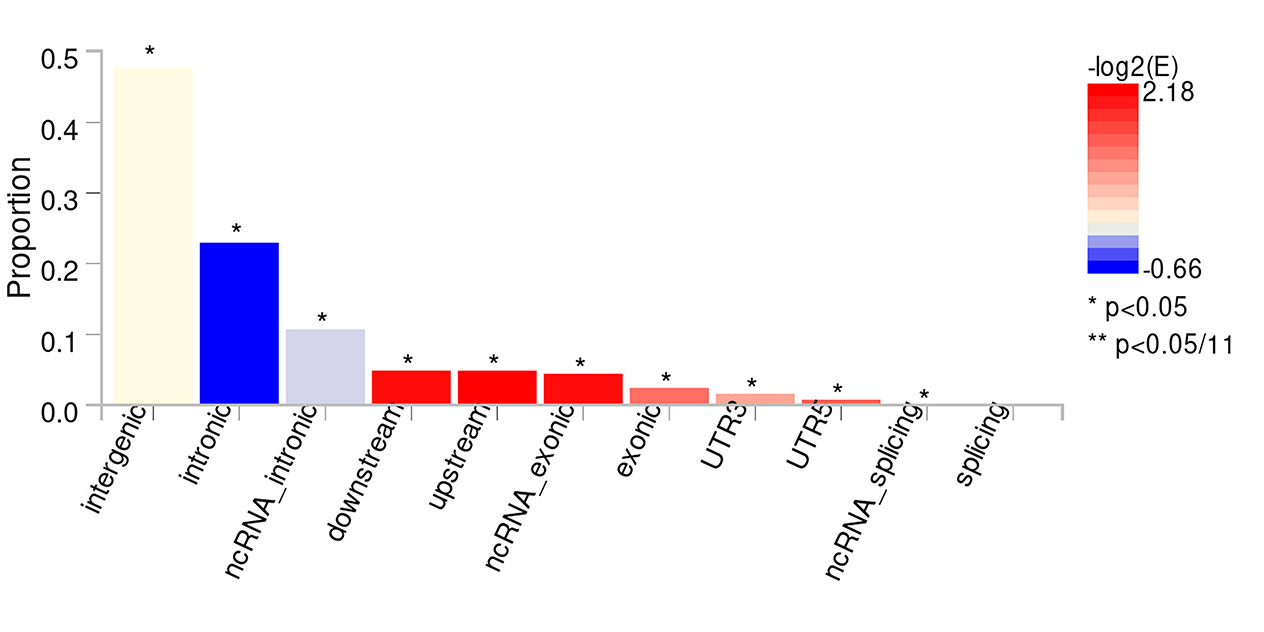

Supplement: Supplementary file 1 [file Image1.jpeg]

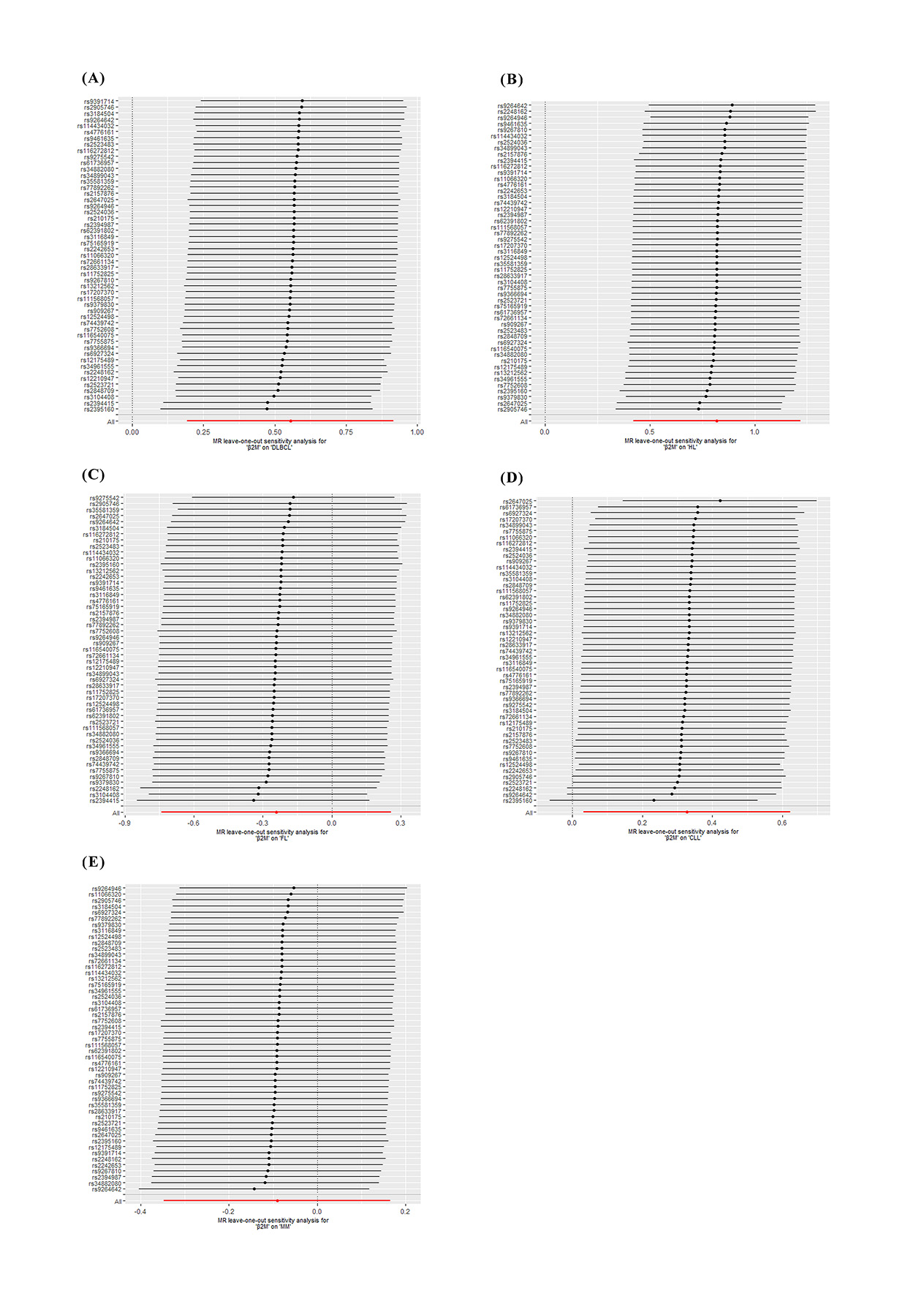

Supplement: Supplementary file 2 [file Image2.jpeg]

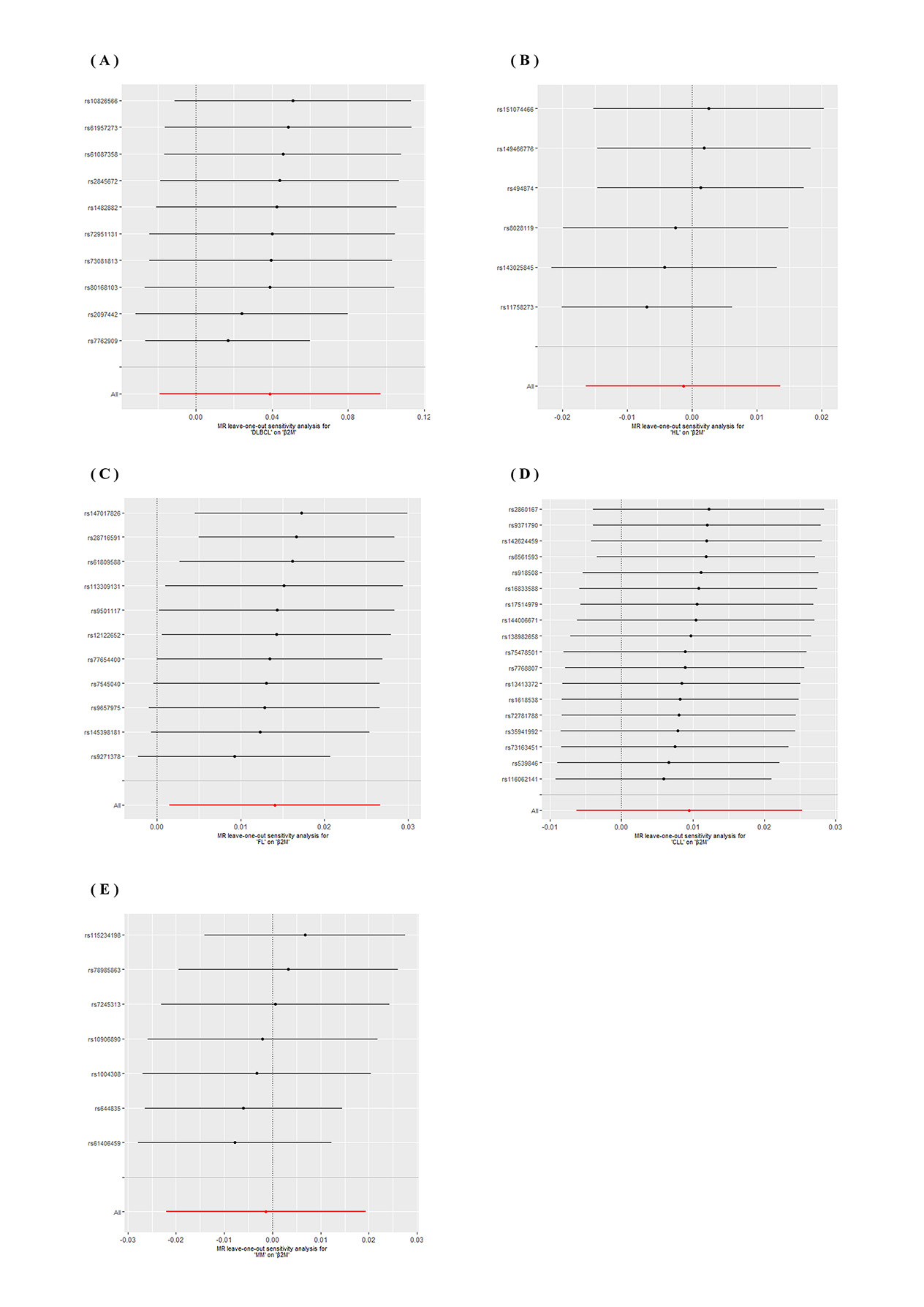

Supplement: Supplementary file 3 [file Image3.jpeg]

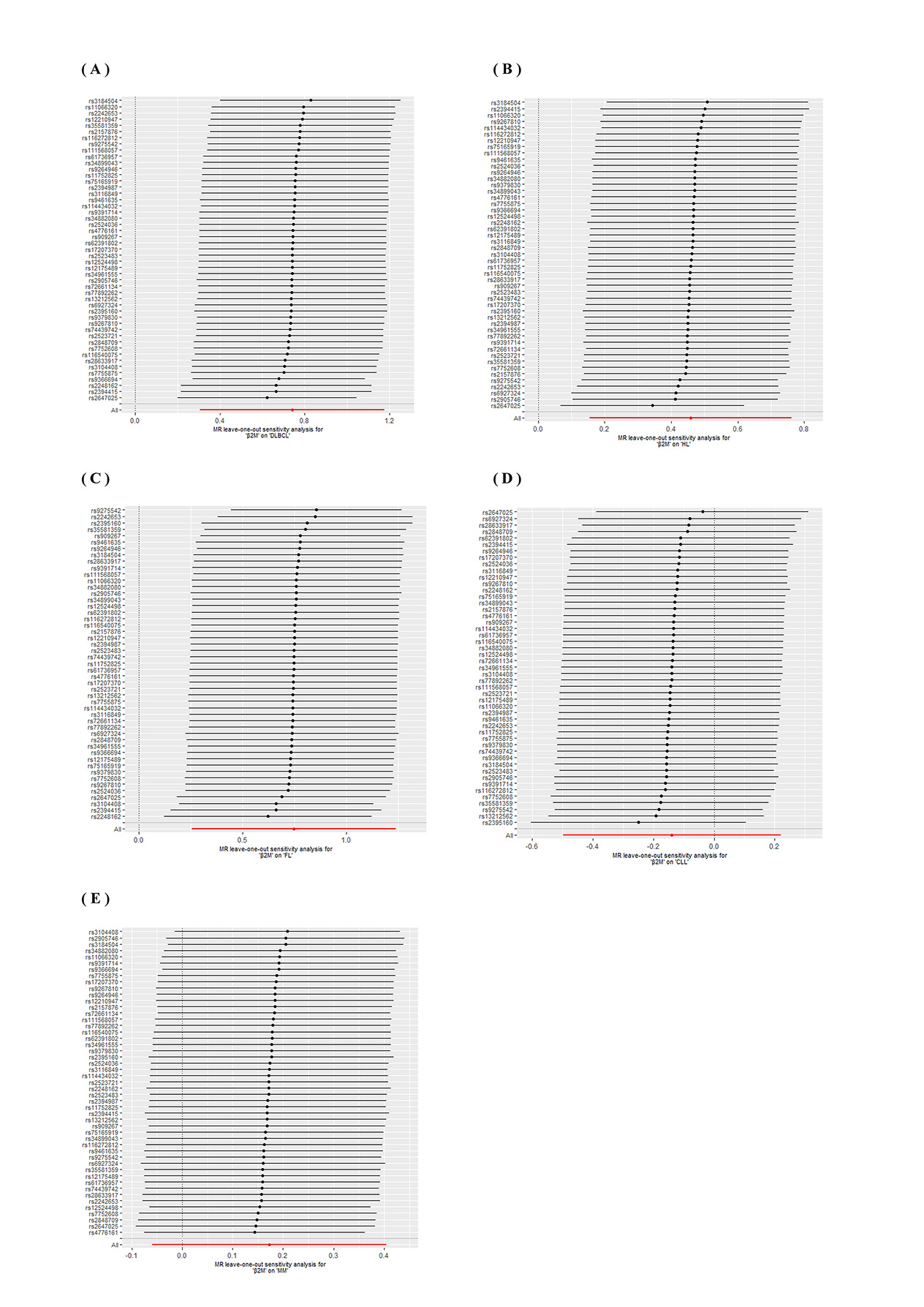

Supplement: Supplementary file 4 [file Image4.jpeg]

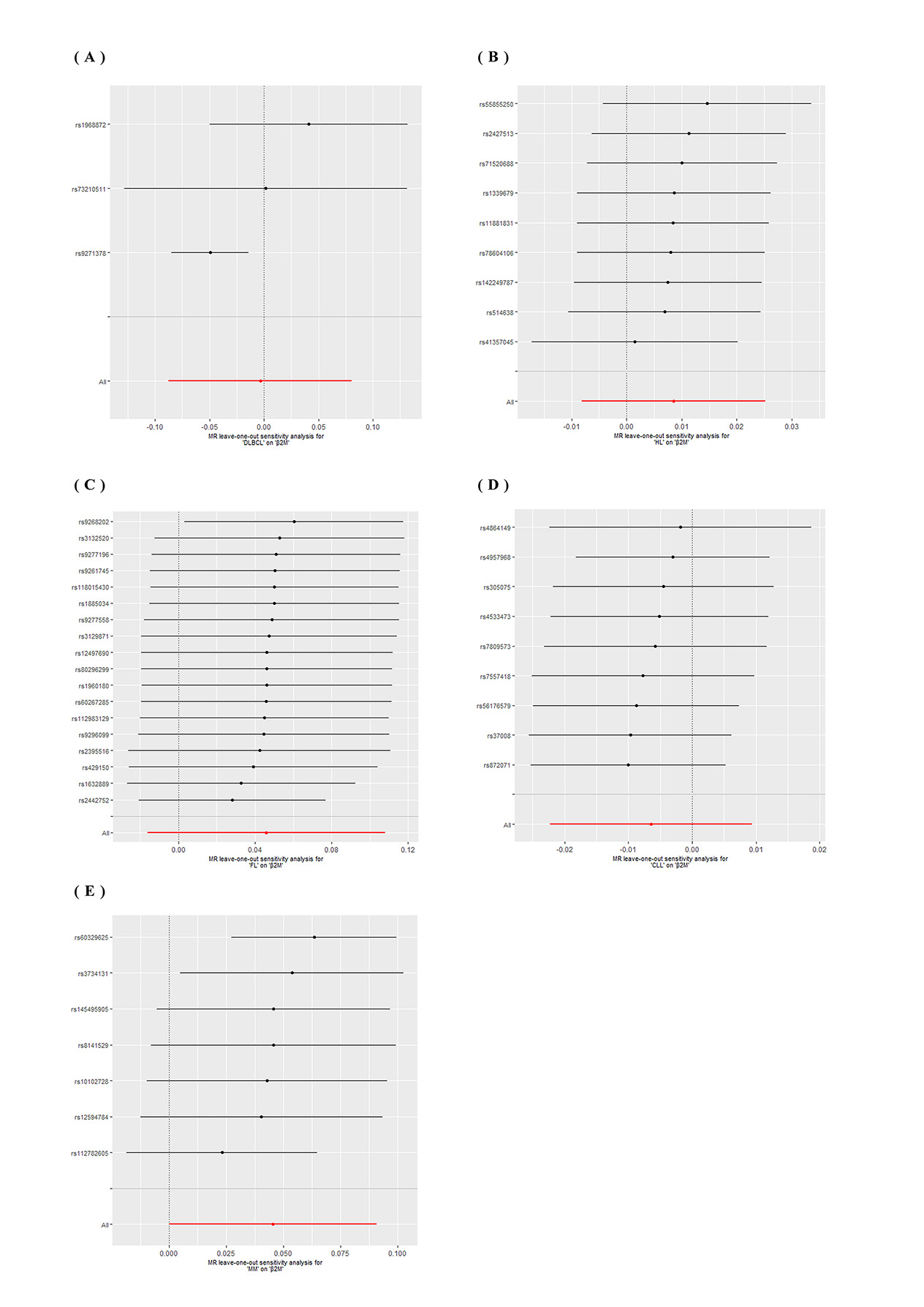

Supplement: Supplementary file 5 [file Image5.jpeg]
